# A framework for scabies control

**DOI:** 10.1371/journal.pntd.0009661

**Published:** 2021-09-02

**Authors:** Daniel Engelman, Michael Marks, Andrew C. Steer, Abate Beshah, Gautam Biswas, Olivier Chosidow, Luc E. Coffeng, Belen Lardizabal Dofitas, Wendemagegn Enbiale, Mosoka Fallah, Elkhan Gasimov, Adrian Hopkins, Julie Jacobson, John M. Kaldor, Fatimata Ly, Charles D. Mackenzie, Jodie McVernon, Matthew Parnaby, Merelesita Rainima-Qaniuci, Oliver Sokana, Dieudonne Sankara, Rie Yotsu, Aya Yajima, Paul T. Cantey

**Affiliations:** 1 Tropical Diseases, Murdoch Children’s Research Institute, Melbourne, Australia; 2 Melbourne Children’s Global Health, Royal Children’s Hospital, Melbourne, Australia; 3 Department of Paediatrics, University of Melbourne, Melbourne, Australia; 4 Clinical Research Department, Faculty of Infectious and Tropical Diseases, London School of Hygiene & Tropical Medicine, London, United Kingdom; 5 Hospital for Tropical Diseases, London, United Kingdom; 6 Communicable Diseases, Neglected Tropical Diseases, WHO Regional Office for Africa, Brazzaville, Congo; 7 Department of Control of Neglected Tropical Diseases, World Health Organization, Geneva, Switzerland; 8 Faculté de Santé de Créteil et Service de Dermatologie, APHP, Hôpital Henri-Mondor, Université Paris-Est, Créteil, France; 9 Research Group Dynamic, EA7380, Faculté de Santé de Créteil, Ecole Nationale Vétérinaire d’Alfort, USC ANSES, Université Paris-Est Créteil, Créteil, France; 10 Department of Public Health, Erasmus MC, University Medical Center Rotterdam, Rotterdam, the Netherlands; 11 College of Medicine, University of the Philippines, Manila, Philippines; 12 Philippine Leprosy Mission, Inc., Manila, Philippines; 13 Department of Dermatovenerology, Bahir Dar University, Medicine and Health Science College, Addis Ababa, Ethiopia; 14 Amsterdam UMC, University of Amsterdam, Amsterdam, the Netherlands; 15 University of Liberia, Monrovia, Liberia; 16 Global Health and Social Medicine, Harvard Medical School, Boston, Massachusetts, United States of America; 17 Division of Country Health Programmes, Malaria, Neglected Tropical Diseases and Other Vector-borne Diseases, WHO Regional Office for Europe, Copenhagen, Denmark; 18 Independent Consultant, United Kingdom; 19 Bridges to Development, Seattle, Washington, United States of America; 20 Public Health Interventions Research Group, Kirby Institute University of New South Wales, Sydney, Australia; 21 Dermatology Unit, EPS Institut d’Hygiéne Sociale de Dakar, Dakar, Senegal; 22 University Cheikh Anta Diop of Dakar, Dakar, Senegal; 23 Task Force for Global Health, Decatur, Georgia, United States of America; 24 Peter Doherty Institute for Infection and Immunity, University of Melbourne and Royal Melbourne Hospital, Melbourne, Australia; 25 Melbourne School of Population and Global Health, University of Melbourne, Melbourne, Australia; 26 Infection Modelling, Murdoch Children’s Research Institute, Melbourne, Australia; 27 Pacific Health Security & Communicable Diseases, Division of Pacific Technical Support, WHO, Suva, Fiji; 28 Ministry of Health, Honiara, Solomon Islands; 29 Tulane School of Public Health and Tropical Medicine, New Orleans, United States of America; 30 School of Tropical Medicine and Global Health, Nagasaki University, Nagasaki, Japan; 31 Department of Dermatology, National Center for Global Health and Medicine, Tokyo, Japan; 32 Division of Communicable Diseases, Medicines, Vaccines and Pharmaceuticals, WHO Regional Office for the Western Pacific, Manila, Philippines; 33 Division of Parasitic Diseases and Malaria, US Centers for Disease Control and Prevention, Atlanta, Georgia, United States of America; 34 Former Medical Officer, Department of Control of Neglected Tropical Diseases, World Health Organization, Geneva, Switzerland; Hitit University, Faculty of Medicine, TURKEY

## Abstract

Scabies is a neglected tropical disease (NTD) that causes a significant health burden, particularly in disadvantaged communities and where there is overcrowding. There is emerging evidence that ivermectin-based mass drug administration (MDA) can reduce the prevalence of scabies in some settings, but evidence remains limited, and there are no formal guidelines to inform control efforts. An informal World Health Organization (WHO) consultation was organized to find agreement on strategies for global control. The consultation resulted in a framework for scabies control and recommendations for mapping of disease burden, delivery of interventions, and establishing monitoring and evaluation. Key operational research priorities were identified. This framework will allow countries to set control targets for scabies as part of national NTD strategic plans and develop control strategies using MDA for high-prevalence regions and outbreak situations. As further evidence and experience are collected and strategies are refined over time, formal guidelines can be developed. The control of scabies and the reduction of the health burden of scabies and associated conditions will be vital to achieving the targets set in WHO Roadmap for NTDs for 2021 to 2030 and the Sustainable Development Goals.

## Introduction

In 2017, scabies was added to the portfolio of the World Health Organization (WHO) Department of Control of Neglected Tropical Diseases (NTDs) because of the burden of scabies and its complications, particularly in areas with limited access to healthcare and because of potential new public health control strategies. To find agreement on common strategies and identify research priorities for a global control strategy, an informal WHO consultation meeting was organized in 2019 [1]. This consultation occurred in the broader context of a new roadmap for NTDs for the period of 2021 to 2030 [2]. Here, we propose a framework for scabies control arising from the expert consultation, within the context of WHO NTD Roadmap. These recommendations represent the view of the expert group, based on current data, and, therefore, the framework is a reasonable basis for piloting of activities, pending formal WHO guidelines.

Scabies is caused by the ectoparasite *Sarcoptes scabiei* var. *hominis*. Transmission generally requires skin-to-skin contact, and zoonotic transmission does not occur. Common scabies infestation typically involves a small number of mites. A hypersensitivity reaction to mites and their products causes itch, which is frequently very severe, and skin lesions of variable severity. Scabies is a strong risk factor for superficial bacterial skin infection (impetigo), which can progress to severe bacterial infections, poststreptococcal glomerulonephritis, and possibly acute rheumatic fever and chronic kidney disease [3].

Scabies occurs in all countries, but most cases occur in low-income and middle-income countries, where overcrowding increases transmission and access to effective treatment is often limited [4]. Significant gaps remain in understanding the global epidemiology of scabies. Modeling studies have estimated a global point prevalence of 100 to 200 million cases of scabies and 455 million annual incident cases [5], but there are very few estimates based on surveys or surveillance. Epidemics can occur in regions with high transmission and in settings of overcrowded living conditions. Institutions, such as aged care facilities, schools, prisons, and hospitals, are common settings for outbreaks, including in high-income settings that otherwise have a low prevalence of scabies [6].

Targeted programs for the public health control of scabies have been piloted in a limited number of sites but have not been implemented at scale. There is strong emerging evidence suggesting that mass drug administration (MDA) can markedly reduce the prevalence of scabies. Effectiveness has been best demonstrated in high-prevalence, island settings [7,8]. There is more limited evidence in non-island populations or lower prevalence contexts [9,10].

## A framework for scabies control

**Fig 1:**
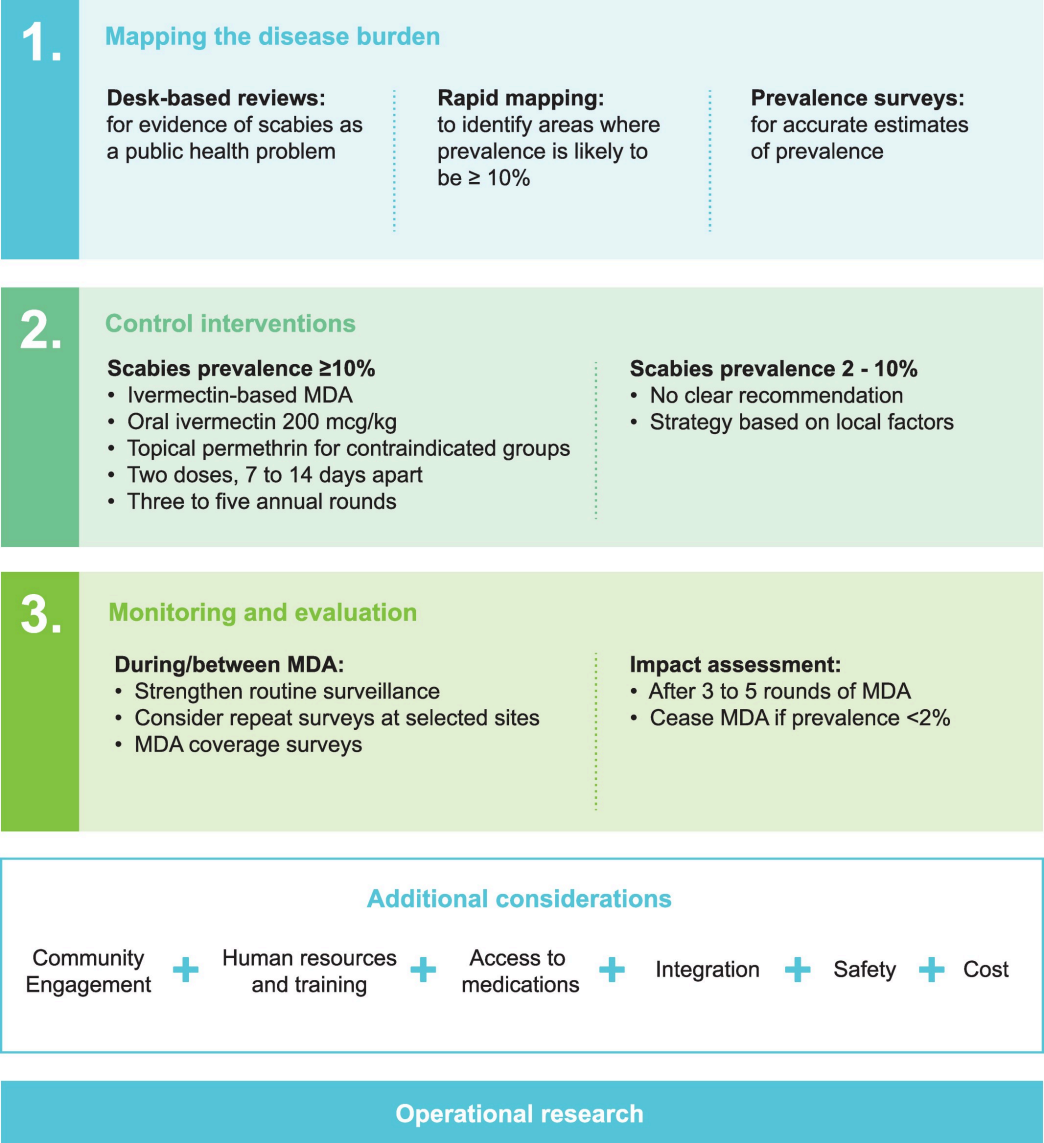
Overview of framework for scabies control. MDA, mass drug administration.

The proposed framework for scabies control includes recommendations for (1) mapping of disease burden; (2) delivery of interventions; and (3) establishing an appropriate monitoring and evaluation framework (Fig 1).

### Mapping the disease burden

Achieving a better understanding of the burden of scabies is impeded by the lack of a “field-friendly” test for diagnosis. Definitive diagnosis is made by microscopic examination of skin scrapings, which is insensitive and impractical in the field. Consensus diagnostic criteria developed by the International Alliance for the Control of Scabies (IACS) categorize diagnosis into 1 of 3 levels of certainty: confirmed scabies (which requires specialized equipment to directly visualize the mite or its products), clinical scabies, and suspected scabies (which rely on clinical signs and history features) [11]. The 2020 IACS criteria could be used as a standard to better define the burden of scabies, as detailed below.

As a prelude to mapping, a desk-based review of routinely collected health data and previous surveys in the country might provide insight into whether scabies is a public health problem and identify likely areas of relatively high burden. Discussions with clinical and managerial health staff are also recommended, as scabies cases are frequently not well captured in routine data.

Two types of survey activities are recommended for different control strategy circumstances. First, rapid mapping, which aims to identify areas where scabies prevalence is likely to be above the 10% threshold for recommending an MDA strategy (see “Control interventions” section below), and second, detailed prevalence surveys, which aim to provide more accurate and longitudinal estimates where these are needed. Community-based, house-to-house sampling across all age groups is recommended as the most robust methodology for both. School-based surveys might be a reasonable alternative where community sampling is not feasible, but this approach requires operational research (Table 1). An implementation unit population of approximately 100,000 to 150,000 people is considered appropriate for both mapping and MDA interventions in most regions, providing a balance between accuracy, detail, and logistical factors, particularly cost.

**Table 1:** Priority research issues for scabies control.

**Medications and safety**
Effectiveness of single-dose ivermectin-based MDA strategy (compared to current 2-dose recommendation)
Safety of ivermectin in currently contraindicated groups (pregnant and breastfeeding women and children weighing <15 kg or <90 cm in height)
Safety of permethrin in infants aged <2 months
Safety of coadministration of ivermectin with medications used in other MDA programs
Efficacy of moxidectin for the treatment and community control of scabies
**Interventions**
Prevalence thresholds for starting and stopping MDA
Optimal number of MDA rounds to achieve sustainable control
Effectiveness of MDA in diverse settings including non-island and urban settings
Effectiveness of control strategies when scabies prevalence is <10% (including targeted MDA, screen and treat, and IDM); comparisons of effectiveness, cost, and feasibility should be made with community MDA
Integration of MDA regimens that use ivermectin for other NTDs to maximize the impact on scabies.
Effect of stopping ivermectin MDA for other NTDs on scabies transmission
Effectiveness of MDA for scabies outbreaks in various settings
**Mapping, monitoring, and evaluation**
Further validate the 2020 IACS criteria, rapid mapping criteria, and training programs for field assessment
Develop monitoring and evaluation methodologies, including sampling, diagnostic tools or needs, and frequency of assessment
Define the relationship between scabies prevalence in schools and in communities
Define the relationship between scabies and impetigo in various settings, including the extent to which MDA for scabies reduces the burden of impetigo
Evaluate different sampling strategies for scabies mapping, including cluster and geospatial sampling designs
Integration of monitoring and evaluation for scabies into existing systems for other diseases
Develop new diagnostic tools (e.g., point of care or rapid diagnostic tests) aligned with programmatic thresholds
Develop strategies to detect recurrence of scabies transmission after cessation of MDA
Monitor for development of resistance of mites to ivermectin and permethrin
**Morbidity and costs**
Determine the impact of scabies MDA on the complications of scabies, including skin and soft tissue infections, invasive bacterial disease, glomerulonephritis, and rheumatic heart disease
Measure impact of scabies and associated impetigo on quality of life, including absenteeism from school and work and broader social costs
Compare cost of scabies control program activities and health-related cost savings and cost-effectiveness of control strategies

IACS, International Alliance for the Control of Scabies; IDM, intensified disease management; MDA, mass drug administration; NTD, neglected tropical disease.

For rapid mapping, diagnosis should be based on a modified, simplified version of the 2020 IACS criteria. A limited skin examination of exposed areas of the limbs for typical lesions of scabies is recommended, conducted by trained primary healthcare workers [12,13].

Prevalence surveys are recommended to provide more detailed regional estimates of prevalence, to advocate for appropriate resources for control, and to monitor prevalence over time, including to assess the impact of control interventions. The 2020 IACS criteria can be used for diagnosis based on clinical assessment in most circumstances. Information about superficial bacterial skin infection (impetigo and infected scabies) should also be collected during rapid mapping and prevalence surveys.

### Control interventions

#### Mass drug administration

Based on existing evidence, pilot control initiatives using MDA are recommended in areas where community prevalence is 10% or higher. A total of 3 to 5 rounds of annual MDA are recommended. The recommended threshold for stopping MDA after these rounds is a community prevalence of <2%. Alternatives strategies to MDA, such as screen and treat (in which the population is screened by clinical assessment and treated only if the exam is consistent with scabies), are likely to be more resource intensive and less effective than MDA, although comparative studies are lacking.

The recommended regimen for MDA is 2 doses of oral ivermectin (200 μg/kg) given 7 to 14 days apart, using directly observed treatment wherever possible. Either weight- or height-based dosing is appropriate. Weight-based dosing is more accurate, while height-based dosing is more practical. Topical treatment is recommended for those for whom ivermectin MDA is currently not approved (pregnant women, lactating mothers in the first week, and children weighing <15 kg or <90 cm height). Evaluation of the safety of ivermectin in these groups is a research priority (Table 1) [14–16]. Permethrin 5% is the preferred topical treatment. Benzyl benzoate can be used where permethrin is unavailable but may be less effective and cause more adverse events. The recommended minimum coverage target is 80% of the total population receiving 2 doses of treatment (either oral or topical).

Environmental measures (e.g., washing linen and clothing) are highly resource intensive and unlikely to add significant benefit to MDA, and, therefore, are not recommended components of a control strategy in low-resource settings. Water, sanitation, and hygiene (WASH) interventions are not effective for scabies but can reduce secondary bacterial infection [17,18].

#### Control strategies below the MDA threshold

MDA is not recommended where the estimated prevalence is <2%. Where the prevalence is between 2% and 10%, there are currently no evidence-based recommendations for the choice of the most appropriate control strategy. The optimal approach should be decided based on the local context and circumstances. Possible strategies include intensified disease management (IDM), screen and treat, or targeted MDA of a high-risk subpopulation. Components of an effective approach might include ensuring scabies treatments are available and affordable where they are needed and enhancing clinical management of scabies in primary care (in particular, ensuring close contacts of an infested patient are treated simultaneously with the patient). Active case finding with appropriate referral or treatment, such as the approach used in screening programs for “skin NTDs,” could be considered [19,20]. Suspected cases of crusted scabies (an uncommon, highly transmissible variant) should be identified and referred for specialized assessment and management of the individual case and their environment. Additional considerations for control are summarized in Table 2. Integration of these strategies into existing health systems and programs is crucial for the success and sustainability of scabies control.

**Table 2:** Additional considerations for scabies control programs.

**Implementation**
Integrate where possible with programs for other NTDs and other health programs, including at stages of mapping, implementation, and surveillance
Engage communities to promote inclusivity, ownership, and sustainability. Use and adapt engagement strategies developed for other NTD programs
Develop and implement packages to train and upskill of health system staff and program managers regarding scabies control issues
Refine existing training packages for assessment of scabies and impetigo for mapping, relevant for local circumstances
**Cost and access**
Ensure that scabies management is included in national essential packages of care as part of Universal Health Coverage
Cost might be a barrier to control because of the higher cost of 2-dose regimens and current absence of a drug donation program
Improve access to low-cost ivermectin and permethrin. This will require local and global advocacy
**Safety**
There is widespread experience using ivermectin and permethrin. Both are considered safe and well-tolerated treatments
Use existing frameworks for safety monitoring and reporting from other NTD programs
A prompt and appropriate response to adverse events is required to maintain confidence in programs

NTD, neglected tropical disease.

#### Control of scabies outbreaks

Ivermectin-based MDA has been effectively used for the control of outbreaks in the community and in closed institutions [6,21]. Further research and consensus are required to define the appropriate methods and thresholds for detecting and declaring scabies outbreaks, the optimal strategy for outbreak control, and criteria for successful control of a scabies outbreak, including when the interventions can be stopped.

### Monitoring and evaluation

An impact assessment should be conducted after completing the planned 3 to 5 rounds, to determine whether MDA should be stopped (i.e., if the prevalence is <2%) or continued longer. More frequent assessment will be required for operational research in initial pilot program sites. Evaluation of impact should assess the burden of scabies, impetigo, and health-related complications. It is also important to monitor for recrudescence of transmission after cessation of MDA [22]. Assessment of cost effectiveness and social impact will be valuable, particularly for initial, pilot programs.

Epidemiologic data on scabies during the program can be collected through a hybrid approach, including sentinel surveillance at selected sites. Methods for impact assessment and surveillance at specific sites should follow the recommendations for prevalence surveys, as described above. Process (e.g., coverage) and outcome (e.g., impact of treatment on prevalence) should be evaluated at some sites, and these should be linked when possible.

Routine collection, analysis, and reporting of data on scabies presentations at healthcare facilities should be strengthened and supported. The results of monitoring and impact assessments should be communicated to frontline workers and affected communities.

## Scabies, Universal Health Coverage, and WHO NTD Roadmap

WHO NTD Roadmap for 2021 to 2030 was endorsed by the 73rd World Health Assembly in November 2020. The roadmap sets global targets, milestones, and strategies to control and eliminate NTDs, as well as crosscutting targets aligned with WHO’s Thirteenth General Programme of Work, 2019–2023 and the Sustainable Development Goals [2,23]. For scabies and other ectoparasites, the proposed targets are the following: (1) the number of countries having incorporated scabies management in the Universal Health Coverage package of care (from 0 in 2020 to 50 in 2025 to all 194 by 2030); and (2) the number of countries using MDA in all endemic districts (from 0 in 2020 to 6 in 2025 to 25 in 2030). The roadmap report notes the critical actions to reach these targets for scabies control, including the development of guidance for mapping and for the implementation of preventive chemotherapy. Opportunities to integrate with other NTD programs where ivermectin is already used (onchocerciasis and lymphatic filariasis) are noted, as is the opportunity to integrate scabies control with approaches to the control of skin NTDs [20,24].

### Next steps

Although there are significant gaps in the evidence upon which to design a scabies control program, there is now sufficient evidence to commence control activities, particularly in the highest prevalence settings. Countries that have identified scabies control as a public health priority can use this framework as a basis for control programs, supported by WHO and organizations such as the World Scabies Program [25]. The implementation of scabies control in these countries will serve as important pilot sites. Priority operational research questions (Table 1) should be addressed alongside implementation, as discussed in detail elsewhere [1,3]. Both ivermectin and permethrin are now included in WHO Model List of Essential Medicines for the treatment of scabies, potentially facilitating prequalification of new manufacturers and suppliers of generic treatments. Work is needed to increase affordable access to these treatments.

## Conclusions

This framework for scabies control is an important step toward addressing an important, unmet global health need. The framework will allow countries to set control targets for scabies as part of national NTD strategic plans and facilitate the development of control strategies using MDA for high-prevalence regions and outbreak situations. It will also assist researchers and partners to tackle the key issues for scabies control. As operational research is completed and pilot projects are scaled up, strategies should be refined and shared. Once sufficient data are available, this framework could develop into formal guidelines to be reviewed through the standard, rigorous WHO process. The successful control of scabies will be vital to achieving the targets set in WHO NTD Roadmap and the Sustainable Development Goals.
